# Diversity and bioactive potentials of culturable heterotrophic bacteria from the surficial sediments of the Arabian Sea

**DOI:** 10.1007/s13205-016-0556-x

**Published:** 2016-11-11

**Authors:** Abdulaziz Anas, Charulatha Nilayangod, C. Jasmin, Saradavey Vinothkumar, P. S. Parameswaran, Shanta Nair

**Affiliations:** CSIR-National Institute of Oceanography, Regional Centre, Kochi, Kerala 682 018 India

**Keywords:** Diversity, Bacteria, antibacterial, Cytotoxic, Oxygen minimum zone, Arabian Sea

## Abstract

Marine sediments accommodate plethora of diverse microorganisms with varying ecological functions. In the present study, we isolated bacteria from surficial sediments of south east Arabian Sea (AS) and evaluated their bioactive potentials. A total of 131 isolates belonging to the phylum: γ-Proteobacteria (63%), Bacillales (34%) and Micrococcaceae (3%) were isolated. Among these, about 40% of the isolates showed the presence of secondary metabolite biosynthetic genes such as PKS or NRPS or both. Organic extracts of nearly 50% of these organisms were cytotoxic to human breast cancer MCF-7 cells and were bactericidal to human pathogens, *Escherichia coli* and *Pseudomonas* sp., while 20–30% of them were bactericidal to *Vibrio* sp. and *Staphylococcus* sp. too. In all, 8 isolates, belonging to *Pseudomonas* spp., *Bacillus* sp. and/or *Lysinibacillus* sp. displayed high level of bactericidal/cytotoxic properties. The study proposes AS sediment as a rich source for microorganisms with prospective bioactive molecules.

## Introduction

Marine microorganisms are exposed to a variety of environmental conditions and are considered as a vast untapped reservoir of metabolic diversity and overwhelming source of novel bioactive compounds. Recent reports of repeated isolation of known metabolites from terrestrial micro/macro organisms indicate that we have almost exhausted this source for novel therapeutics (Daniel 2004; Debbab et al. 2010; Sponga et al. 1999). Contrary to this, several novel bioactive metabolites have recently been reported from different marine organisms. It is also possible that many of these compounds, initially isolated from marine invertebrates, could be produced by associated microorganisms (Mehbub et al. 2014). It is believed that 10–20% of the bacteria isolated from marine environment have biotechnological (Armstrong et al. 2001; Lemos et al. 1985; Zheng et al. 2005) or medicinal (antimicrobial, cytotoxic, antioxidant, antiangeogenesis, antidiabetic, etc.) (Debbab et al. 2010; Thakur et al. 2005; Zheng et al. 2005) properties. Mostly, these reports are based on the bioprospecting studies of microorganisms associated with mangroves, sponges, sea weeds, etc., leaving aside the larger portion of marine environment, i.e., sediments and other extreme environments almost untouched (Beedessee et al. 2015; Santos-Gandelman et al. 2014).

Oxygen minimum zones (OMZ) are subsurface oceanic regions characterized by lethargic circulation of oxygen-poor waters (<8 µM), high primary productivity, intense denitrification and high oxygen demand due to decaying sinking organic matter (Banse et al. 2014). Nearly 59% of world OMZ are being reported from the water column of Arabian Sea at depths of 100–1000 m (Banse et al. 2014). The intensity of OMZ may extend up to the coastal water (<20 m), in response to climatic changes, upwelling, river discharge and natural or anthropogenic fertilization (Paulmier and Ruiz-Pino 2009). OMZ alters the chemical composition of the surrounding environment and also induce stress on the organisms (Childress and Sieibel 1998; Schneider and Bush-Brown 2003). Previous studies on OMZ have concentrated primarily on the diversity of microorganisms in these waters/sediments (Bryant et al. 2012; Divya et al. 2010, 2011) and their functional role in nitrogen cycle (Paulmier and Ruiz-Pino 2009). Though OMZ constitute only about 0.1% of the total oceanic volume, about 30–40% of the total oceanic nitrogen loss is estimated to occur within it. It is believed that this denitrification is mediated by denitrifying and anaerobic ammonia oxidizing bacteria (anammox) (Codispoti et al. 2001; Lam et al. 2009; Woebken et al. 2008). Recent studies have also reported microbial suphate reduction and sulphur oxidation in OMZ (Canfield et al. 2010). However, the bioactive potential of these microorganisms have not been studied yet in detail. An earlier study from our group had reported the enzymatic diversity of bacteria isolated from the sediments of Arabian sea OMZ region (Divya et al. 2010). In the present study, sediment samples were collected from perennial or seasonal OMZ regions of AS and reported the diversity and bioactive properties of associated heterotrophic bacteria.

## Methodology

### Sample collection, separation of microorganisms

**Fig. 1:**
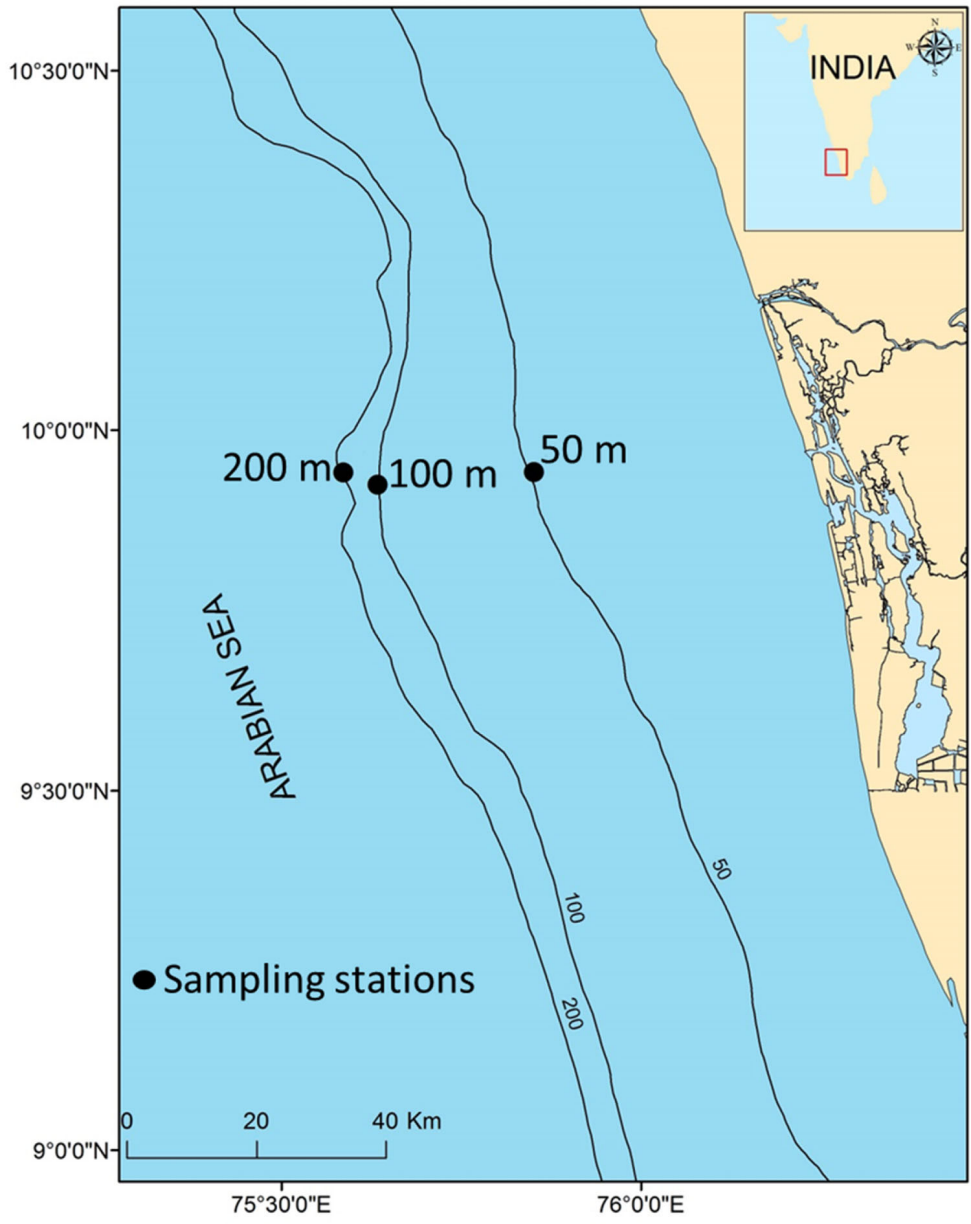
Map showing sampling locations across Kochi Transect of Arabian Sea

Sediment samples collected from three different depths (50, 100 and 200 m) of Cochin transect of Arabian Sea (Fig. 1) during FORV Sagar Sampada cruise (No 289) was used for the isolation of bacteria. Aliquots of sediment samples (100 g) from Grab sampler were removed aseptically into sterile polypropylene bottles and maintained at 4 °C until further analysis. Total microorganisms were separated from sediment samples following differential centrifugation with slight modifications (Fieseler et al. 2006). Briefly, sediment samples were mixed with sterile seawater and disintegrated mechanically using a mortar and pestle. The bacteria released into seawater were separated by filtering sequentially through nylon mesh (ca. 200 µ), 70 and 30 µ filter cups. The remaining sediment particles in the filtrate was separated by repeated centrifugation at 100*g* for 10 min, and the bacteria were separated by centrifugation at 12,000*g* for 30 min (Biofuge Stratos, Heraeus Germany). The presence of microorganisms was confirmed microscopically after staining an aliquot with DAPI. The microorganisms were preserved in artificial seawater containing 20% glycerol at −80 °C.

### Isolation and purification of Microorganisms

The microorganisms separated from sediment as mentioned above were serially diluted up to 10^6^ and 100 µl from each dilution was spread over the surface of Oligo (trypton 0.05; yeast extract 0.005; sodium glycerol phosphate 0.01 and agar 1.5%), Basal (trypton 0.5; yeast extract 0.1; glucose 0.2 and agar 1.5%), Starch casein nitrate (starch 1.0; casein 0.03; K_2_PO_4_ 0.2; KNO_3_ 0.2; MgSO_4_·7H_2_O 0.005; FeSO_4_·2H_2_O 0.002; CaCO_3_ 0.001 and agar 1.5%) and starch casein (starch 1.0; casein 0.1 and agar 1.5%) medium. All the media were prepared in 50% sea water and adjusted to pH 7.6 ± 0.2. The plates were incubated at 28 ± 2 °C up to 3 weeks and morphologically different colonies were isolated and purified at every 48 h.

### Identification of microorganisms

Based on morphological characteristics, 40 representative isolates were selected and identified by 16S rRNA gene sequencing. Genomic DNA was extracted from overnight grown cultures following standard phenol–chloroform method, and the quality of DNA was checked on 0.8% agarose gel (Sambrook and Russel 2001). The 16S rRNA gene of the bacteria (~1465 bp) was amplified using universal primers [27F: AGAGTTTGATC(AC)TGGCTCAG and 1492R: GGTTACCTTGTTACGACTT] (Lane 1991) in a 25 µl reaction volume containing 1 µl DNA (50–100 ng), 1 µl each of primers (10 pmol µl^−1^), 2.5 µl 10× Taq polymerase buffer (NEB, Canada), 0.5 U Taq DNA polymerase (NEB, Canada) and 200 µM of each dNTPs (NEB, Canada). PCR cycling conditions maintained were as follows: initial denaturation at 95 °C for 2 min, followed by cycle denaturation at 95 °C for 40 s, annealing at 55 °C for 40 s, extension at 72 °C for 1.5 min for a total of 30 cycles and a final extension for 10 min at 72 °C. PCR products were purified using Nucleo-pore Genetix brand Sure Extract PCR clean-up/Gel extraction kit (Genetix Biotech, India) and used as a template for sequencing PCR using internal primer 1090R [GCTCGTTGCGGGACTTAACC] (Amann et al. 1995). Sequencing PCR was done with ABI PRISM Big Dye terminator ready reaction mix (Life Technologies, USA). The cycle extension products were purified following ethanol/EDTA/sodium acetate precipitation. The products were analysed on an Applied Biosystems ABI 3730xl DNA analyzer. Sequence data obtained were analysed and edited using Sequencher V4.10.1 (GeneCodes, USA).

The sequences were analysed in SILVA rRNA gene database project (https://www.arb-silva.de/ngs) (Quast et al. 2013). The sequences were aligned using the SILVA incremental aligner against the SILVA SSU rRNA SEED. The sequences with more than 98% similarity to each other were clustered into a single OTU and the longest sequence in each cluster was selected as representative OTUs. The nearest neighbours of representative OTUs were selected from NCBI by nucleotide BLAST search. Phylogenetic tree of representative OTUs was constructed using MEGA (5.5) software. The sequences of representative OTUs and those with bioactive potentials were submitted to NCBI (Accession No. KT818688 to KT818696, KX442647 to KX442650).

### Screening of isolates for the presence of NRPS and PKS genes

One hundred and thirty-one isolates from sediment samples were screened for the presence of secondary metabolite biosynthetic genes such as Polyketide synthase (PKS) and non-ribosomal peptide synthetase (NRPS) genes. DNA was extracted from overnight grown cultures following standard phenol–chloroform method, and the quality of DNA was checked on 0.8% agarose gel (Sambrook and Russel 2001). The NRPS (700–800 bp) and PKS (1200–1400 bp) genes of the bacteria were amplified using primer combinations of A3F (GCSTACSYSATSTACACSTCSGG)-A7R (SASGTCVCCSGTSCGGTAS) and K1F(TSAAGTCSAACATCGGBCA)-M6R(CGCAGGTTSCSGTACCAGTA), respectively (Ayuso-Sacido and Genilloud 2005). The PCR reaction was carried out in a 25 µl reaction volume as mentioned above. PCR cycling conditions maintained were as follows: initial denaturation at 94 °C for 2 min, followed by cycle denaturation at 94 °C for 30 s, annealing at 59/55 °C (respectively, for NRPS and PKS gene) for 30 s, extension at 72 °C for 2 min for a total of 35 cycles and a final extension for 10 min at 72 °C. The PCR products segregated on 0.8% agarose gel were stained with SYBR green and visualized in a gel documentation system (BioRad, USA).

### Preparation of organic extracts

Organic extract of 131 isolates was prepared using ethyl acetate as solvent. Bacterial isolates for the production of secondary metabolites were prepared in basal medium. Briefly, 100 μl of bacterial inoculum (1.0 OD at 600 nm) prepared in basal medium was transferred into fresh medium (100 ml) and incubated at 28 ± 2 °C for 7 days in a rotary shaker maintained at 100 rpm. The supernatant from 7-day-old bacterial culture was mixed with equal volume of ethyl acetate and extracted by shaking at 180 rpm for 1 h. The ethyl acetate phase was separated and concentrated in a rotary evaporator under vacuum at 40 °C. The residue thus obtained was weighed, stock solutions prepared in DMSO and maintained at 4 °C till further use.

### Cytotoxic and antibacterial properties

The cytotoxicity of extracts was tested in triplicate in MCF7 breast cancer cell line using 3-(4,5-dimethylthiazole-2-yl)-2,5-diphenyltetrazolium chloride (MTT) assay. Briefly, MCF7 cells were cultured up to 50% confluence in a 96-well microplate containing Dulbecco’s modified Eagle’s medium (DMEM) supplemented with 10% foetal bovine serum (FBS). The cells were washed copiously with phosphate-buffered saline (PBS), and the medium was exchanged with DMEM (10 ml) containing test compounds (20 ppm). Cells exposed to DMEM or DMSO alone were maintained in separate wells as control. The cells were incubated for 24 h at 37 °C and subjected for MTT assay following standard protocol. Briefly, cells treated with test compounds were supplemented with 50 µl of MTT solution (5 mg ml^−1^) prepared in PBS and kept for incubation under dark at 37 °C for 3 h. Subsequently, the viability of the cells was measured as a function of reduction of MTT to insoluble formazan by mitochondrial dehydrogenase enzyme of healthy cells. Formazan crystals were dissolved in Dimethyl Sulphoxide and the absorbance was recorded at 570 nm using a microplate reader (Biotech, USA).

The antibacterial property of the organic extracts against four isolates of multiple drug resistant bacteria, *Staphylococcus aureus*, *Pseudomonas aeruginosa*, *Vibrio cholera* and *Escherichia coli*, was tested using standard disc diffusion assay. Briefly, the 6 mm diameter paper discs impregnated with 100 µl test compound (20 ppm) were placed over the surface of a Muller Hinton agar plate swabbed with test organisms. Paper discs impregnated with 100 µl DMSO were also used as control. The plates were incubated for 24 h at 28 ± 2 °C and the formation of clear zones around the discs was considered as the positive indication of inhibitory activity. The zone of inhibition around the discs was recorded after 24 h using Hi Antibiotic Zone Scale (Himedia, India).

## Results and discussion

**Fig. 2:**
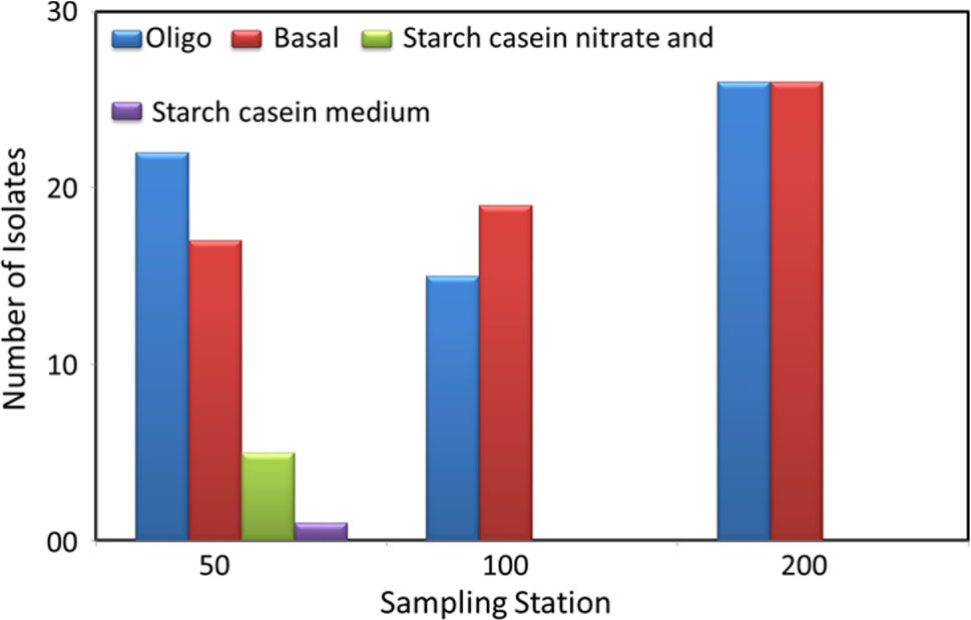
Bar diagram showing the number of bacteria grown on different medium

A total of 131 bacteria were isolated from sediment samples of AS in four different media. Higher number of bacterial colonies were observed on Oligo and basal medium, while only few colonies were found on starch-based medium (Fig. 2). The oligo and basal media were prepared in seawater and they differed in the concentration of nutrients. These two mediums used simple carbon and nitrogen sources, while starch casein and starch casein nitrate medium used more complicated nutrient sources. Different bacteria in marine environment differ in their function and accordingly their nutrient preference also varies. It is a widely accepted practice that in such cases, medium with different nutrient composition are used to retrieve maximum number of isolates.

**Fig. 3:**
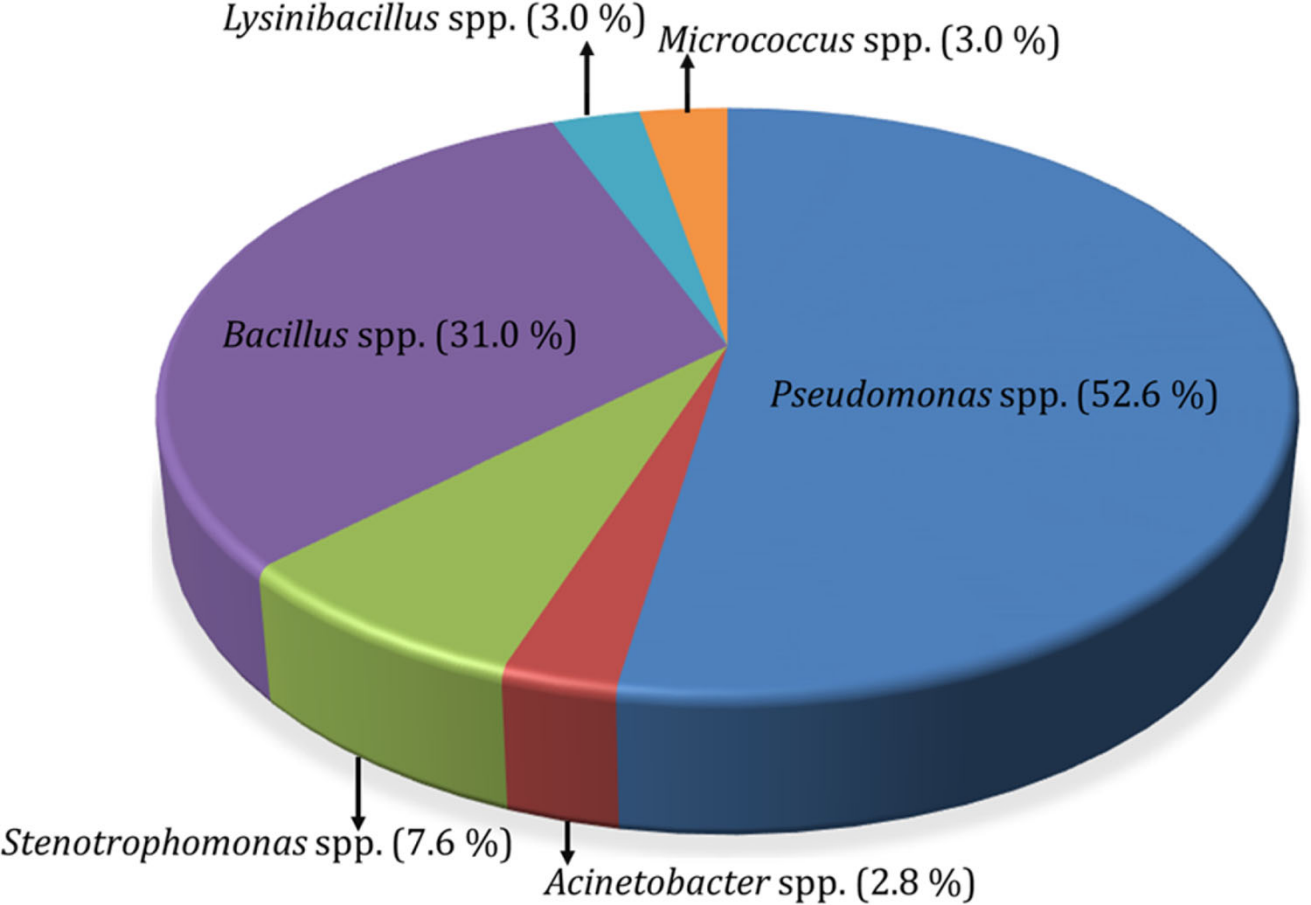
Relative abundance of different OTUs of bacteria isolated from AS. The pie diagram is prepared based on Krona plot (Ondov et al. 2011) generate by analysis of sequences in SILVA rRNA gene database project (https://www.arb-silva.de/ngs)

**Fig. 4:**
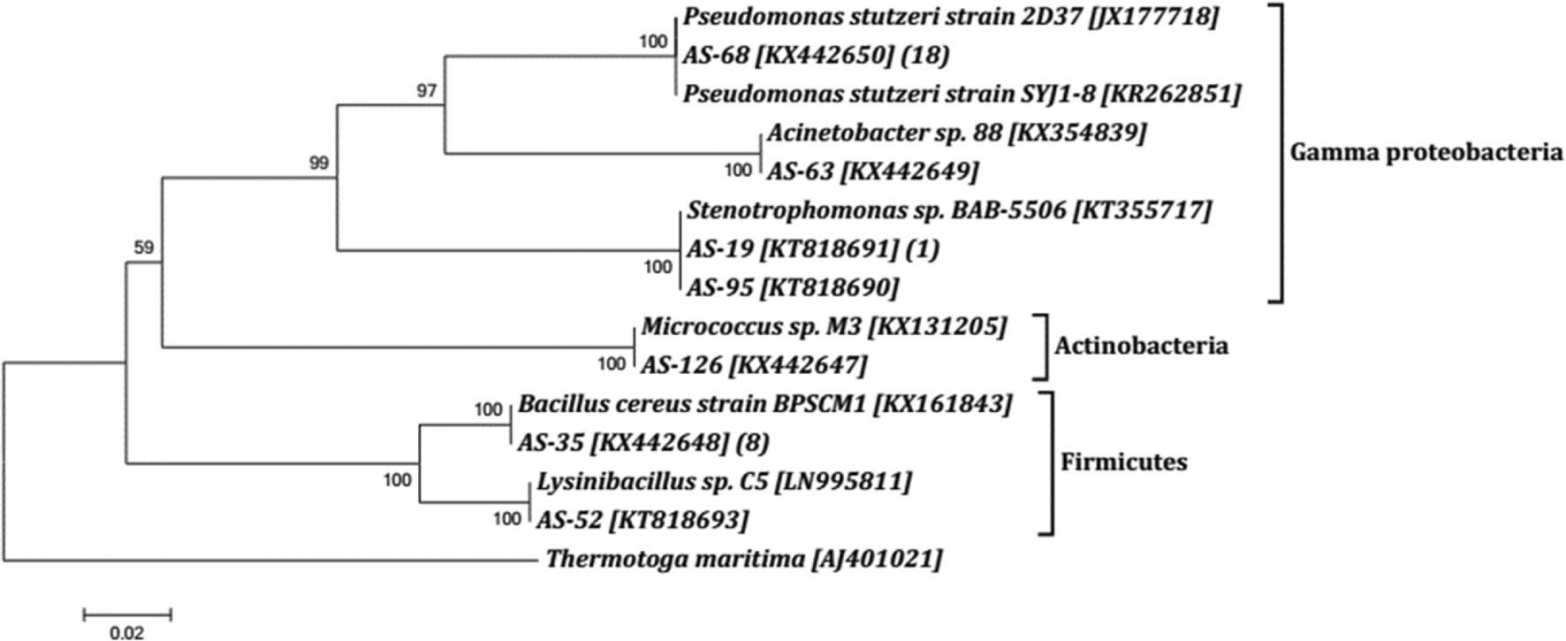
Rooted neighbour-joining phylogenetic tree based on 16S rRNA gene sequence of heterotrophic bacteria isolated from surficial sediment of AS. The numbers at each nodes are percentages indicating the levels of bootstrap support based on neighbour joining analysis of 1000 resampled data sets. Number in bracket indicates the number of isolates in each OTUs

Forty representative morphotypes of these isolates were identified using 16S rRNA gene sequencing. Upon sequence analysis, they were clustered into seven OTUs, the relative abundance of which is represented in Fig. 3. Majority of the isolates belonged to γ-Proteobacteria, among which 53% were *Pseudomonas* sp. (1 OTU), 8% were *Stenotrophomonas* sp. (2 OTUs) and 3% were *Acinetobacter* sp (Fig. 4). The second dominant group of bacteria isolated from AS sediment were Bacillales, with 31% *Bacillus* sp. (1 OTU) and 3% *Lysinibacillus* sp. (1 OTU) (Fig. 4). A minor group of isolates represented 3% of *Micrococcus* sp. (1 OTU). The previous reports from deep sea sediments of Pacific Ocean also reported the dominance of γ-Proteobacteria (Delong et al. 1997; Kato et al. 1996; Wang et al. 2004). The dominance of Firmicutes and γ-Proteobacteria in sediments of AS at 200, 500 and 1000 m depth of Goa coast was reported earlier (Divya et al. 2010). Divya et al. (2011) also confirmed the dominance of Proteobacteria in AS sediments by culture-independent clone library approach. Although the diversity of bacteria from different marine environments are reported, an ecological interpretation, including which group of bacteria are dominant in different environment (like oligotrophic, OMZ, vents, etc.), is limited. Such studies from soil environments indicated a positive correlation between the carbon mineralization rate and Proteobacteria (Fierer et al. 2007; Ge et al. 2010). If this concept is taken, the AS sediment being a burial ground of organic carbon (Dale et al. 2015) may support the proliferation of Proteobacteria.

**Fig. 5:**
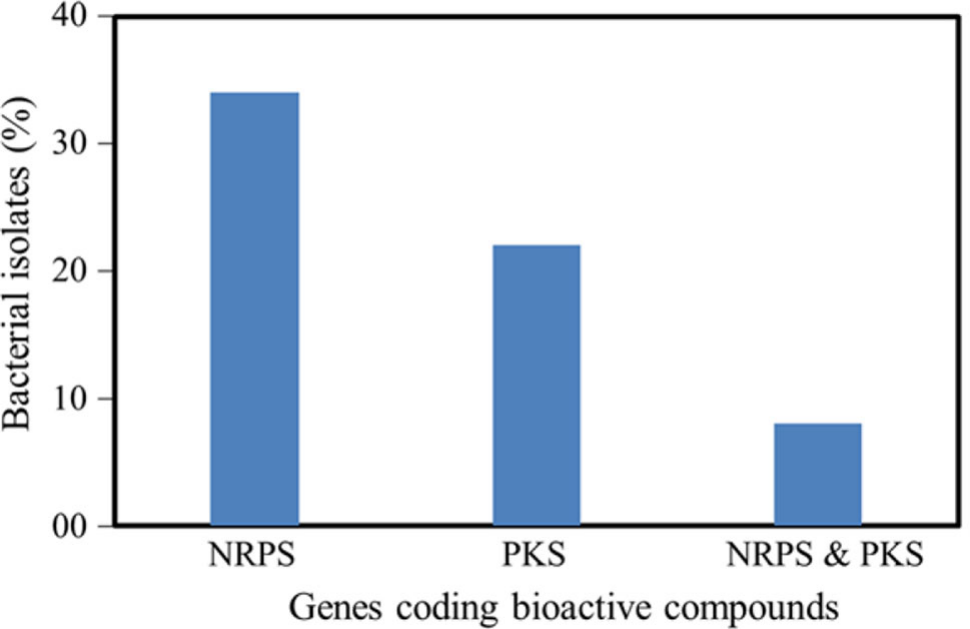
Number of isolates showing the presence of NRPS, PKS genes

Although metagenomic approaches can provide more resolved status on diversity of bacteria, we used culture-dependent methods as screening for bioactive potential was one of the objective of the present study. Here the bioactive potentials of all the isolates were studied at different levels; using molecular and cellular assays. It was observed that ~40% of isolates showed the presence of either NRPS or PKS genes. NRPS and PKS genes were present in 34 (26%) and 22 (17%) isolates, respectively (Fig. 5). Interestingly, the presence of both NRPS and PKS genes was observed among 8 isolates (6%). The presence of PKS and NRPS genes are reported in different microorganisms associated with sponges and marine sediments (Bull and Stach 2007; Schirmer et al. 2005). NRPS and PKS enzymes involved in the early phases of secondary metabolite biosynthesis pathways and synthesize common intermediary chemical structure, which then modified into diverse secondary metabolites (Wohllenben et al. 2012).

**Fig. 6:**
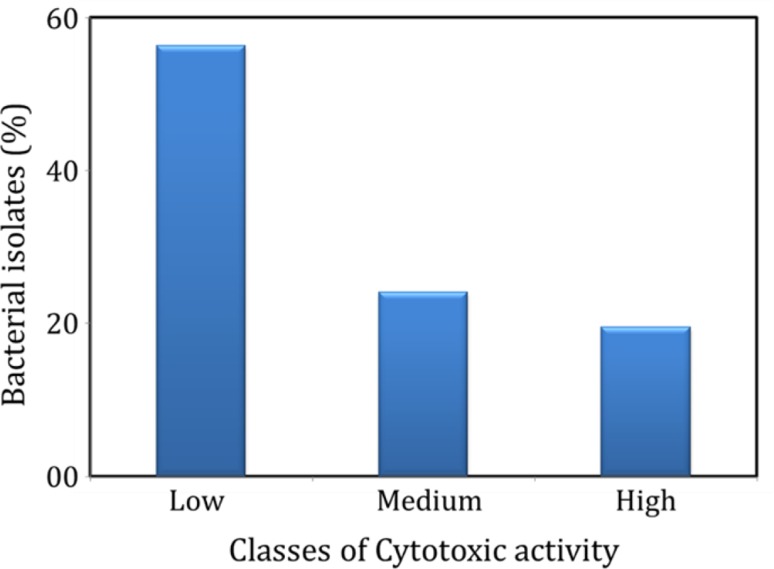
Distribution of bacteria isolated from AS sediments, based on the cytotoxic activity of their ethyl acetate fraction in breast cancer MCF7 cells. The organisms were classified based on their cytotoxicity into low (0–10% cytotoxicity), medium (10–30% cytotoxicity) and high (>30% toxicity) cytotoxic groups

**Fig. 7:**
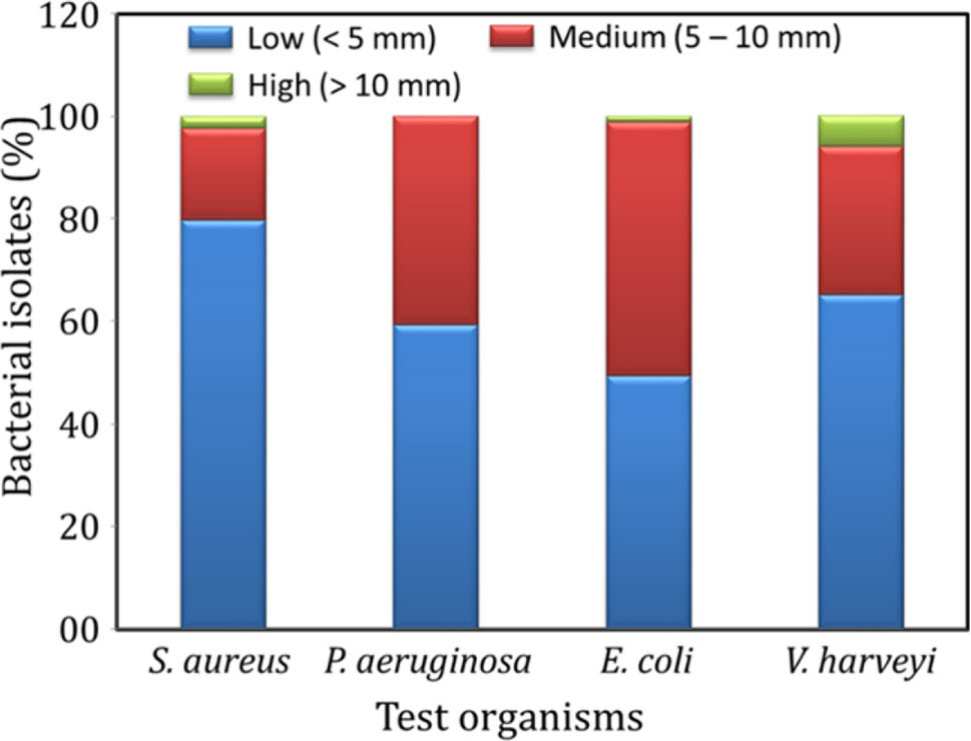
Histogram showing the classification of bacteria isolated from AS sediment, based on the antibacterial activity of their ethyl acetate fraction against *Staphylococcus aureus*, *Pseudomonas aeruginosa*, *Escherichia coli* and *Vibrio cholerae*. The organisms were classified based on their antibacterial activity into low (<5 mm clearing zone), medium (5–10 mm clearing zone) and high (>10 mm clearing zone) activity groups

PCR screening for PKS and NRPS genes is advantageous as it permits the high-throughput screening for potential isolates, even if they do not produce the secondary metabolites under given culture conditions. However, the secondary metabolites synthesized through pathways other than PKS and NRPS may not be reflected in this assay. Though laborious, conventional bioassays permits the direct detection of secondary metabolites produced through different pathways. Therefore, in the present study, we used a combination of PCR and conventional bioassays. In MTT assay, nearly 50% isolates showed medium or higher level of cytotoxicity in MCF7 breast cancer cell line (Fig. 6). Antibacterial activity was also shown by many isolates, while the intensity of activity varied between test organisms (Fig. 7). The organic extracts from 40 to 50% of the isolates showed medium or high level of antibacterial activity against *E. coli* and *Pseudomonas* sp., while the number of isolates with potential activity against *Vibrio* sp. and *Staphylococcus* sp. were 30 and 20%, respectively.

These results indicate that sediments of the AS could be a promising source for novel bioactive molecules. The number of bacteria with bioactive potentials in sediments of AS are much higher compared to that reported from sediment of intertidal zone of China (Zheng et al. 2005) and bacteria associated with marine eukaryotes (Penesyan et al. 2009). Meanwhile, the results are comparable to that of sponge-associated bacteria; for example, more than 50% of bacteria isolated from the sponge *Haliclona* sp. and 34% from *Irciniidae* sp. showed antibacterial properties (Ana et al. 2013; Thomas et al. 2010).

We ranked eight isolates as prospective ones for further investigation on novel bioactive potentials based on their high cytotoxicity (i.e. >40%) and antibacterial properties (Table 1). All the selected isolates except AS 8 had either NRPS or PKS gene. The NRPS and PKS gene cluster present in AS 8 may be novel and hence could not be amplified using the primer used in the study. Novel NRPs and PKS gene clusters have been reported recently from marine bacteria (Machado et al. 2015; Sun et al. 2016). *Pseudomonas* spp. AS 8, 106 and 70 displayed promising activity against MCF 7 cell line (41, 50 and 45% mortality, respectively). The extract from the first two isolates was also moderately active against *E coli* and *Staphylococcus* sp. respectively, while that of strain AS 70 was inactive against these bacterial pathogens. The bioactive potentials of marine *Pseudomonas* sp. and their application in aquaculture have been reported earlier (Chythanya et al. 2002; Pai et al. 2010). Many compounds have been extracted from marine organisms with cytotoxic and antibacterial activities (Zhao et al. 2013), while compounds with selective killing of prokaryotic or eukaryotic cells are rarely reported. Our results indicate that AS 70 is a potential source of bioactive molecules which can selectively kill the eukaryotic cells. Our studies also underline the importance of AS sediment as a source of bacteria with novel bioactive potentials.

**Table 1:** Consolidated table showing the bioactive potentials of eight selected isolates

Activity	Isolate no (AS−)							
	8	19	25	52	70	81	95	106
Presence of gene								
NRPS	−	+	+	+	+	−	+	+
PKS	−	−	+	−	−	+	−	+
Cytotoxicity (dead cell, %)	41	39	44	43	45	48	43	50
Antibacterial activity (diameter of clearing zone in mm)								
*Staphylococcus* sp.	0	12	7	6	0	6	0	6
*Pseudomonas* sp.	0	7.5	0	0	0	7	0	6
*E. coli*	6	8	0	0	0	6.5	0	0
*Vibrio* sp.	0	21	0	6	–	7	6	0

Our study proposes sediments of AS with rich source of diverse bacteria as potential source for bioprospecting novel bioactive molecules. The organic extract from nearly 50% of the 131 isolates retrieved from sediments of AS showed cytotoxicity to human breast cancer cells MCF 7 and was bactericidal to human pathogens, *E. coli* and *Pseudomonas* sp. Eight isolates of *Pseudomonas* spp., *Bacillus* sp. *and Lysinibacillus* sp. showed exceptional cytotoxic/antibacterial properties. Further large-scale bioprospecting of bacteria from sediments of AS focused on the identification, purification and characterization of novel bioactive molecules will be appreciated.
